# Transient Expression in HEK-293 Cells in Suspension Culture as a Rapid and Powerful Tool: SARS-CoV-2 N and Chimeric SARS-CoV-2N-CD154 Proteins as a Case Study

**DOI:** 10.3390/biomedicines11113050

**Published:** 2023-11-14

**Authors:** Thailin Lao, Omar Farnos, Alexi Bueno, Anays Alvarez, Elsa Rodríguez, Julio Palacios, Kathya Rashida de la Luz, Amine Kamen, Yamila Carpio, Mario Pablo Estrada

**Affiliations:** 1 Center for Genetic Engineering and Biotechnology, Animal Biotechnology Department, Havana 10600, Cuba; thailin.lao@cigb.edu.cu (T.L.);; 2 Department of Bioengineering, McGill University, Montreal, QC H3A 0E9, Canada; omar.farnosvillar@mcgill.ca (O.F.); amine.kamen@mcgill.ca (A.K.); 3 Process Development Department, Center of Molecular Immunology, Havana 11600, Cubajulio@cim.sld.cu (J.P.); katiar@cim.sld.cu (K.R.d.l.L.)

**Keywords:** transient expression, HEK-293, suspension culture, serum-free, SARS-CoV-2, N protein

## Abstract

In a previous work, we proposed a vaccine chimeric antigen based on the fusion of the SARS-CoV-2 N protein to the extracellular domain of the human CD40 ligand (CD154). This vaccine antigen was named N-CD protein and its expression was carried out in HEK-293 stably transfected cells, grown in adherent conditions and serum-supplemented medium. The chimeric protein obtained in these conditions presented a consistent pattern of degradation. The immunization of mice and monkeys with this chimeric protein was able to induce a high N-specific IgG response with only two doses in pre-clinical experiments. In order to explore ways to diminish protein degradation, in the present work, the N and N-CD proteins were produced in suspension cultures and serum-free media following transient transfection of the HEK-293 clone 3F6, at different scales, including stirred-tank controlled bioreactors. The results showed negligible or no degradation of the target proteins. Further, clones stably expressing N-CD were obtained and adapted to suspension culture, obtaining similar results to those observed in the transient expression experiments in HEK-293-3F6. The evidence supports transient protein expression in suspension cultures and serum-free media as a powerful tool to produce in a short period of time high levels of complex proteins susceptible to degradation, such as the SARS-CoV-2 N protein.

## 1. Introduction

Since the emergence of severe acute respiratory syndrome coronavirus 2 (SARS-CoV-2) in December 2019, the dashboard of the WHO has reported more than 700 million confirmed cases and almost 7 million deaths caused by this virus globally (https://covid19.who.int/, accessed on 5 September 2023). Although most of the approved vaccines are based on the SARS-CoV-2 Spike (S) protein or specifically on the receptor binding domain (RBD) of this protein [1,2], many scientists have proposed the use of the SARS-CoV-2 N protein as an important target for vaccine development [3,4,5,6,7,8]. Indeed, recent studies have demonstrated the role of the N protein in modulating innate and adaptive immunity [9] and how N-specific humoral responses could mediate antibody-dependent cellular cytotoxicity (ADCC) and help control SARS-CoV-2 infection [8,10]. Basic studies related to aspects other than S proteins of betacoronavirus are also important to characterize their structures, biophysical properties and biological functions.

In a previous work, we proposed a vaccine antigen based on the fusion of the SARS-CoV-2 N protein to the extracellular domain of the human CD40 ligand (hCD40L or CD154), as molecular adjuvant [11]. This vaccine antigen was named N-CD protein and its expression was carried out in human embryonic kidney 293 (HEK-293) cells due to the high transfectivity, rapid growth and human-like post-translation modifications provided by these cells [12,13]. The N-CD protein was produced in adherent conditions and serum-supplemented medium using lentiviral vectors, and presented a consistent pattern of degradation. Despite that, the immunization of mice and monkeys with this chimeric protein was able to induce a high N-specific IgG response with only two doses. With the aim of evaluating the adjuvant effects of soluble CD154, we decided to obtain the SARS-CoV-2 N protein in HEK-293 cells in the same culture conditions as mentioned above. However, the N protein showed a similar degradation pattern, probably produced by the presence of proteases in the serum used to supplement the culture medium [14,15]. A possible solution to this problem is the elimination of this compound via the adaptation of the recombinant cell lines to a suspension culture and serum-free media. Nevertheless, this is a complex process that can take up to 6 months and, in some cases, a decrease in protein expression has been observed after the adaptation [16,17]. To test our hypothesis, we decided to express both proteins in suspension cultures and serum-free media using transient expression in HEK-293SF cells (clone HEK-293SF-3F6) at different scales. This system has been successfully used to express SARS-CoV-2 proteins (RBD and N protein) [18,19,20,21] and other complex proteins [22,23,24,25]. The results showed almost no degradation of the N and N-CD proteins. This encouraged us to generate N-CD-expressing stable transfected clones and adapt them to suspension culture and serum-free media to potentially meet the regulatory requirements for N-CD expression in a future production setting. Similar results were obtained in a batch culture and simulation of a perfusion culture of adapted N-CD-expressing cell clones. The evidence support transient protein expression in suspension culture and serum-free media as a powerful tool to study and produce proteins in a short period of time compared with the generation of stable cell clones, in conditions resembling the final production settings at industrial scale. It also seems an appropriate system for the rapid manufacturing of complex proteins susceptible to degradation, as observed with the SARS-CoV-2 N and N-CD proteins.

## 2. Materials and Methods

### 2.1. Biological Reagents

**Figure 1:**
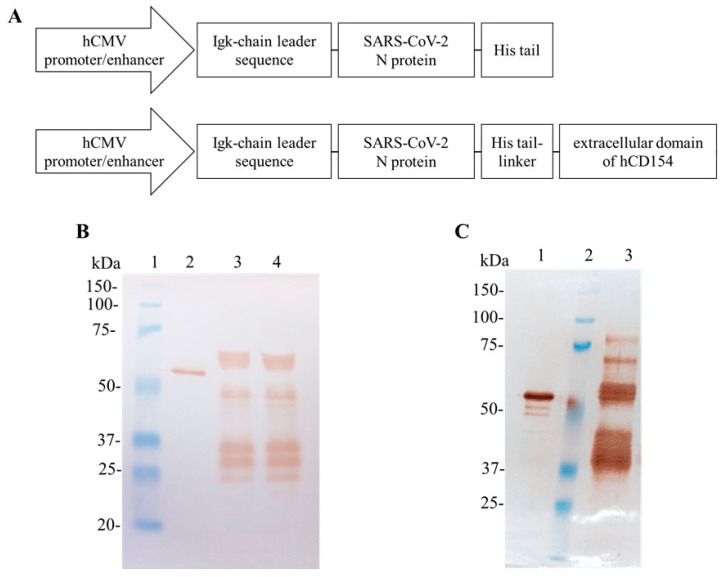
SARS-CoV-2 N and N-CD proteins produced in adherent conditions by recombinant HEK-293 cells. (**A**) Schematic diagram of the expression cassettes pl6twblast-CMV-N-His and pl6twblast-CMV-N-CD. (**B**,**C**): Western blot analysis under reducing conditions. SARS-CoV-2 N protein produced in *E. coli* was used as positive control. The antibody CBSSNCoV-1-HRP was used for protein detection. (**B**) Cell culture supernatant harvested after 5 days of culture in DMEM/F12 medium. Recombinant cells were seeded in 24-well plates at 0.3 × 10^6^ cells/well in 1 mL of DMEM/F12-FBS culture medium. Twenty-four hours later, the cell monolayer was carefully washed with PBS and 1 mL of DMEM/F12 medium was added per well. Lane 1: Precision Plus ProteinTM All Blue Prestained Standard; lane 2: positive control; lane 3: cell culture supernatant of HEK-293-N-100 cells; lane 4: cell culture supernatant of HEK-293-N-200 cells. (**C**) N-CD protein purified previously in the laboratory with IMAC. Lane 1: positive control; lane 2: Precision Plus ProteinTM All Blue Prestained Standard; lane 3: purified N-CD protein. Protein visualization was carried out with DAB.

SARS-CoV-2 N protein (2-419 aa) obtained in *Escherichia coli* (CIGB, Sancti Spiritus, Cuba), with a molecular weight of 45 kDa, was used as positive control in SDS-PAGE and Western blots, as well as a standard for ELISA. Purified N-CD protein obtained from a cell pool in adherent conditions was previously obtained in our laboratory (Figure 1) [11].

### 2.2. Mammalian Cell Lines and Cell Culture Conditions

Human embryonic kidney cells (HEK-293, ATCC CRL-1573) were used for transient expression and transduction with lentiviral vectors bearing the N gene and recombinant protein expression. The lentiviral particles were produced using HEK-293FT (ATCC PTA-5077) as packing cells. We also used two different N-CD-expressing HEK-293 cell pools previously obtained in our laboratory [11], HEK-293-N-CD-50 cells and HEK-293-N-CD-100 cells. All these cell lines were cultured in Dulbecco’s Modified Eagle’s Medium/Ham’s Nutrient Mixture F-12 (DMEM/F12) medium (Gibco, USA) supplemented with 10% Fetal Bovine Serum (FBS, Capricorn, Australia) (DMEM/F12-FBS) or not, taking into account the procedural requirements. Cells were maintained in a static humidified incubator at 5% CO_2_ and 37 °C. 

For transient expression in suspension culture and serum-free media, an HEK-293 serum-free cell line was used. This cell line is derived from HEK-293SF suspension cells (specifically clone HEK-293SF-3F6) and were obtained from a GMP-grade master cell bank [26]. The cells were grown in chemically defined and animal-component-free HyCell TransFx-H medium (HyClone, Cytiva, Marlborough, MA, USA), with 6 mM Gibco GlutaMAX (Fisher Scientific, Canada) and 0.1% Kolliphor Poloxamer 188 (Millipore-Sigma, Canada). An antibiotic (Penicillin-Streptomycin, Gibco, USA) was only added for culturing cells in the 3 L bioreactor. The shake flasks were maintained in shaking conditions at 37 °C, 120 rpm and 5% CO_2_ in an incubator shaker (Infors HT, Switzerland).

### 2.3. SARS-CoV-2 N Plasmid Construction and Lentivirus Production

For N plasmid construction, the expression cassette (hCMV promoter/enhancer + murine Ig κ-chain leader sequence + N gene + 6x-Histidine tail) was amplified via PCR from the plasmid pl6twblast-CMV-N-CD [11], and cloned into the lentiviral plasmid pl6twblast, which encodes a blasticidin deaminase gene as selection marker. The following forward and reverse oligonucleotide primers were used: 5′-ATATATCCCGGGGCGCGCGTTGACATTGATTATTG-3′ and 5′-TCAATGGTGATGGTGATGATGCCCGGCCTGAG-3′, respectively. The PCR product was digested with *XmaI* and ligated into the plasmid pl6twblast previously digested with the restriction enzymes *XmaI* and *EcoRV*. The plasmid obtained was named pl6twblast-CMV-N-His (Figure 1). 

For lentiviral packaging, three helper plasmids from the third generation HIV-1-based lentiviral packaging system were used (ViraPowerTM Lentiviral Packaging Mix, Invitrogen, Carlsbad, CA, USA).

Lentivirus bearing the SARS-CoV-2 N gene (LV-N) was produced via transfection of HEK-293FT. The cells were transfected with the plasmid pl6twblast-CMV-N-His and the helper plasmids from the third-generation HIV-1-based LV packaging system using PEI (Linear, MW 160 kDa, PolyScience, Niles, IL, USA), as described by [27]. The LV-N was purified through a Lenti-X™ Concentrator kit (Clontech, USA) following manufacturer’s instructions. The LV-N stock single-use aliquots were stored at −80 °C until use. The Lv-N stock was quantified using an ELISA for detection of HIV p24 capsid protein (DAVIH-Ag p24, LISIDA, Mayabeque, Cuba). 

### 2.4. Transduction of HEK-293 Cells and Generation of SARS-CoV-2 N-Protein-Expressing Cell Pools

The HEK-293 cells were transduced using LV-N and polybrene, at a final concentration of 10 µg/mL, to improve the transduction process. The procedure implemented was similar to the method described by [27,28]. Two different MOI were used: 100 and 200. A negative control of transduction was included comprising cells that were cultured under selection conditions with blasticidin but were not incubated with LV-N. Six hours after exposure to LV-N, 500 µL of selection culture medium 2X (DMEM/F12 supplemented with 20% FBS and 16 µg/mL blasticidin) was added to a final concentration of 10% FBS and 8 µg/mL blasticidin. Transduction procedures were repeated a second time as described above. After 24 h of incubation at 37 °C in 5% CO_2_, the metabolized culture medium was carefully removed and 1 mL of selection culture medium (DMEM/F12-FBS and 8 µg/mL blasticidin) was added to transduced and negative control cells. The selection culture medium exchange was performed every 48–72 h for 21 days.

### 2.5. Assessment of SARS-CoV-2 N-Protein Expression and Degradation in the Cell Culture Supernatant of Recombinant HEK-293 Cells Cultured in Adherent Conditions

The SARS-CoV-2 N-expressing HEK-293 cell pools obtained (HEK-293-N-100 cells and HEK-293-N-200 cells) were used to study the degradation kinetics of the SARS-CoV-2 N protein in cell culture supernatant of adherent HEK-293 cells. The cells were seeded at 0.3 × 10^6^ cells per well in 1 mL of DMEM/F12-FBS in triplicates in 24-well plates. Six plates were seeded in the same conditions, one per day of the kinetic study. After an overnight incubation at 37 °C in 5% CO_2_, the cell monolayer presented 70–80% cellular confluence. The metabolized culture medium was removed, cells were gently rinsed with 1 mL of DMEM/F12 medium and 1 mL of DMEM/F12 medium was carefully added per well. The plates were incubated at 37 °C in 5% CO_2_ and samples of cell culture supernatant were collected every 24 h. Cell density and viability were determined with a hemocytometer and the trypan blue exclusion method. The cell culture supernatant was analyzed with ELISA and Western blot. 

### 2.6. Production of the SARS-CoV-2 N and N-CD Proteins in Cell Suspension Culture

HEK-293SF-3F6 cells were seeded in 1 L shake flasks at 1.5 × 10^6^ cell/mL in 200 mL of HyCell TransFx-H medium. For transfection, 1 µg of plasmid DNA (pl6twblast-CMV-N-His or pDisplay-CMV-N-CD) was used per 1 million cells, using PEIPro^®^ transfection reagent (Polyplus Transfection, USA) at a ratio of (3:1) (PEI:DNA) (*w*/*w*). The cell suspension was harvested 72 h post transfection and centrifuged at 2500× *g* for 20 min at 4 °C. The clarified culture supernatant was stored at −20 °C and later thawed for protein analysis and purification. Afterwards, proteins were also produced in 1 L bioreactors (Applikon Biotechnology, Delft, The Netherlands). Bioreactors were seeded at a viable cell density of 0.35 × 10^6^ cells/mL in HyCell TransFx-H medium with working volumes of 1100 mL and 900 mL for the production of the N and N-CD proteins, respectively. For the N-CD protein, another run in a 3 L bioreactor (Applikon Biotechnology, The Netherlands) was also implemented. The bioreactor was inoculated at a viable cell density of 0.2 × 10^6^ cells/mL in a working volume of 2600 mL using HyCell TransFx-H medium supplemented with antibiotic (Pen-Strep) (Gibco, USA). In all cases, the transfection was carried out when the cells reached viable cell densities between 1 and 1.7 × 10^6^ cells/mL. For 1 L and 3 L bioreactors, PEI (Linear, MW 25 kDa, PolyScience, USA) at a ratio of (3:1) (PEI:DNA) (*w*/*w*) was used. 

For the three independent runs, the bioreactor control unit automatically controlled the dissolved oxygen (DO) concentration and pH. The DO concentration was maintained at 40% air saturation with the injection of pure oxygen and continuous surface aeration of 12.5 mL/min. The pH was set at 7.15 and fluctuations around this value were controlled via the addition of NaHCO_3_ (90 g/L) or the injection of CO_2_ into the headspace. The 1 L bioreactors were equipped with a single marine impeller and the 3 L bioreactor with a double marine impeller. In both cases, the agitation was kept at 100 rpm. Approximately every 24 h, samples of cell culture were collected from each bioreactor and cell density and viability were determined in a Vi-CELL XR cell counter (Beckman Coulter Life Sciences, Brea, CA, USA). The cell suspension was harvested 72 h post transfection and centrifuged at 2500× *g* for 20 min at 4 °C. The clarified culture supernatant was stored at −20 °C and later thawed for analysis and protein purification. 

### 2.7. Cell Cloning of the HEK-293-N-CD-50 and HEK-293-N-CD-100 Cell Pools via Limiting Dilution

Cell cloning of the HEK-293-N-CD-50 and HEK-293-N-CD-100 cell pools was performed via limiting dilution in 96-well plates [28,29]. To measure the expression levels of the N-CD-expressing HEK-293 cell clones, an experiment was performed in 24-well plates in static conditions. The cell clones and pools were seeded at 0.3 × 10^6^ cells/well in 1 mL of DMEM/F12-FBS medium in triplicate. Plates were incubated at 37 °C in 5% CO_2_ and after 7 days cell culture supernatant was harvested to quantify N-CD protein expression levels using ELISA.

### 2.8. Batch Culture of the N-CD-Expressing Cell Clones Cultured in Suspension Culture and Serum-Free Medium

The N-CD-expressing cell clones were adapted to serum-free medium and suspension culture as described by [27,28]. The growth kinetics of N-CD-expressing cell clones adapted to CDM4HEK293 medium and suspension culture in agitated conditions were performed in batch culture using 250 mL shake flasks or 2 L roller bottles. In the 250 mL shake flasks, cells were seeded at 0.4 × 10^6^ cells/mL in 90 mL of CDM4HEK293 medium and incubated in a warm chamber (37 °C) in a shaker (130 rpm) without CO_2_. The roller bottles were seeded at 0.35 × 10^6^ cells/mL in 500 mL of CDM4HEK293 medium and placed vertically in an incubator (Infors HT, Switzerland) at 37 °C with 5% CO_2_ in shaking conditions (120 rpm). All the experiments were performed in duplicate. Every 24 h, samples of cell culture were collected and cell density and viability were determined with a hemocytometer and the trypan blue exclusion method. Cell culture supernatant was collected to quantify N-CD-expression levels with ELISA. In the case of 250 mL shake flasks cultured without CO_2_, they were incubated at 37 °C with 5% CO_2_ in static conditions for 2 or 3 h, after taking samples for cell counting and before to be incubated again in the conditions described above. 

The maximum growth rate (μ_max_) in h^−1^ was determined as the slope of the following, Equation (1):

VCD(t) = VCD(t_0_) + µ_max_ × t (1)

where VCD(t_0_) and VCD(t) are viable cell density at times 0 and t in the exponential phase of cell growth, respectively. 

### 2.9. Simulation of Perfusion Culture of the N-CD-Expressing Cell Clones Cultured in Suspension Culture and Serum-Free Medium

Perfusion culture in a stirred-tank bioreactor of N-CD-expressing cell clone was simulated in 250 mL shake flasks. The cells were seeded at 0.4 × 10^6^ cells/mL in 90 mL of CDM4HEK293 medium and incubated at 37 °C with 5% CO_2_ in shaking conditions (120 rpm) in an incubator (Infors HT, Bottmingen, Switzerland). The experiment was performed in duplicate. Before starting perfusion culture simulation, the cells were maintained in batch culture conditions until exponential phase and maximum growth rate were reached. Afterwards, the perfusion culture started with dilution rates of 0.27 and 0.189 vessel volume exchanged per day (vvd), for flask1 and flask 2, respectively, until reaching 1 vvd. To calculate the dilution rate, the cell-specific perfusion rate (CSPR) was taken into account. In these experiments, CSPR was fixed to 0.15 nL/cell/day. This value was previously determined considering the performance of the cells in the culture medium. Every 24 h, samples of cell culture were collected and cell density and viability were determined with a hemocytometer and the trypan blue exclusion method. After cell counting, the dilution rate was calculated using the following, Equation (2):

D = CSPR × VCD(2)

where D is dilution rate (vvd), CSPR is cell-specific perfusion rate (fixed value of 0.15 nL/cell/day) and VCD is viable cell density (cell/mL).

The D value was multiplied by the volume of cell culture, in this case 90 mL, to determine the volume of cell suspension to be extracted from each flask. This cell suspension was centrifuged at 300× *g* for 5 min. The supernatant was collected and stored at −20 °C for protein analysis and purification. The cellular pellet was resuspended in the same volume of fresh CDM4HEK293 medium and returned to the corresponding flask to a final volume of 90 mL. 

### 2.10. Protein Purification

For the purification of the SARS-CoV-2 N and N-CD proteins in HyCell TransFx-H medium, the clarified cell culture supernatant was concentrated and buffer-exchanged to equilibrium conditions (300 mM NaCl, 50 mM NaH_2_PO_4_, 20 mM imidazole and pH 7.4) via tangential flow filtration (TFF) using a 10 kDa hollow fiber filtering module (Spectrum, USA). The concentrated and equilibrated supernatant was filtered through 0.45 µm membrane using bottle tap vacuum filters (Thermo Fisher, USA), then loaded into a HisTrapTM HP-5 mL affinity column with AKTATM Avant 25 system (Cytiva, USA). Briefly, the column was washed with 5 CV of equilibrium buffer and later with 2.5 CV of washing buffer (300 mM NaCl, 50 mM NaH_2_PO_4_ and 90 mM imidazole, pH 7.4). The elution was executed with 4 CV of elution buffer (300 mM NaCl, 50 mM NaH_2_PO_4_ and 250 mM imidazole, pH 7.4). A final washing step with 2.5 CV (300 mM NaCl, 50 mM NaH_2_PO_4_ and 250 mM imidazole, pH 7.4) was executed to elute all residual proteins and to re-equilibrate the column before the next run. The working flow rate was 2.5 mL/min at room temperature. The purified SARS-CoV-2 N and N-CD proteins were buffer-exchanged to storage buffer (PBS, 2 mM MgCl_2_ and 2% sucrose, pH 7.5) using Amicon centrifugal filters (10 kDa cut-off, Merck Millipore, USA) or PD10 desalting column (GE Healthcare, UK), respectively.

### 2.11. Protein Quantification with ELISA

The quantification of the SARS-CoV-2 N and N-CD proteins present in the cell culture supernatant of the cells cultured in adherent conditions was carried out with sandwich ELISA, as described by [11]. The *E. coli* SARS-CoV-2 N protein was used to prepare a standard curve (5 to 0.156 µg/mL) for protein quantification. Samples were analyzed in triplicate. 

### 2.12. SDS-PAGE and Western Blot

Protein samples were analyzed in 10% SDS-PAGE as described by Laemmli [30] under reducing conditions. In some cases, the samples were previously concentrated using Amicon^®^ Ultra-2 mL centrifugal filters (Merck-Millipore, USA) or the method of trichloroacetic acid (TCA) and sodium deoxicolate. After running, proteins were stained with Coomassie Brilliant Blue (G-250 or R-250). Proteins separated via gel electrophoresis were electro-transferred to a nitrocellulose membrane (GE Healthcare, Germany or Bio-Rad, USA). A blocking step was performed by incubating the membrane with 5% skim milk in washing buffer (PBS, 0.05% Tween 20, pH 7.4) for 1 h at room temperature in shaking conditions or overnight at 4 °C. Subsequently, the membrane was rinsed for 5 min with washing buffer and this step was repeated 3 times. A 6x-His Tag monoclonal antibody conjugated to horseradish peroxidase (HRP) (His.H8, MA1-21315-HRP, Thermo Fisher Scientific, Waltham, MA, USA), at a dilution 1:1000 in blocking buffer (5% skim milk in washing buffer), was used for the detection of the 6x-Histidine tail present in both proteins. A system based on primary and secondary antibodies diluted in blocking buffer, 6x-His Tag monoclonal antibody (1:1000, His.H8, MA1-21315, Thermo Fisher Scientific, USA) and HRP conjugated-goat anti-mouse IgG (H+L) polyclonal (1:10,000, 115-035-003, Jackson Immuno Research, West Grove, PA, USA), were also used for this purpose. For epitope-specific SARS-CoV-2 N-protein detection, the membrane was exposed to an HRP-conjugated anti-SARS-CoV-2 N-protein monoclonal antibody (CBSSNCoV-1-HRP, CIGB-Sancti Spiritus, Cuba) at a dilution 1:2000 in PBS. A SARS-CoV-2 N protein mouse monoclonal antibody (1:1000, clone ID: OTI4B9, TA814506, ORIGENE, USA) as primary antibody and the same HRP conjugated-goat anti-mouse IgG (H+L) polyclonal as secondary antibody were also incubated with the membrane to detect both proteins based on their SARS-CoV-2 N protein moiety. All the antibodies were incubated at room temperature for 1 h in shaken conditions. The membrane was washed again and substrate was added for reaction development. Protein detection and visualization were carried out through two different methodologies: 3,3′-diaminobenzidine (DAB) (Applichem, Germany) and ECL detection system (GE Healthcare, UK or Bio-Rad, USA), following manufacturer’s instructions. 

### 2.13. Statistical Analysis

Data were analyzed using Excel software (Microsoft Office Professional Plus 2019). The results are expressed as mean ± standard deviation (SD).

## 3. Results

### 3.1. Production of the SARS-CoV-2 N Protein in Adherent HEK-293 Cells

The N protein obtained in the cell culture supernatant of HEK-293 cells transduced with lentiviral vectors, at MOI of 100 and 200, showed a pattern of multiple bands in Western Blot analyses under reducing conditions (Figure 1). The anti-SARS-CoV-2 N-protein monoclonal antibody recognized one wide band migrating above 50 and below 75 kDa, which corresponds to the N protein, as well as groups of protein bands between 50 and 25 kDa for the samples of culture supernatant corresponding to the two MOI evaluated (Figure 1B). The expected molecular weight for the recombinant N protein is 48.2 kDa. The N protein expression kinetics in the cell culture supernatant from both cell pools was assayed (Figure S1). The protein band between 50 and 75 kDa was only detected after 98 h (Figure S1). The HEK-293-N-200 cells were selected to produce the N protein for further purification due to its higher expression levels compared with HEK-293-N-100, as determined with ELISA and Western blot (Figure S1). This pattern resembled the one observed for the N-CD protein expressed by an HEK-293-N-CD stable cell pool cultured in adherent conditions (Figure 1C).

### 3.2. Production of SARS-CoV-2 N and N-CD Proteins through Transient Expression in Suspension Conditions and Serum-Free Medium Using Shake Flasks

**Figure 2:**
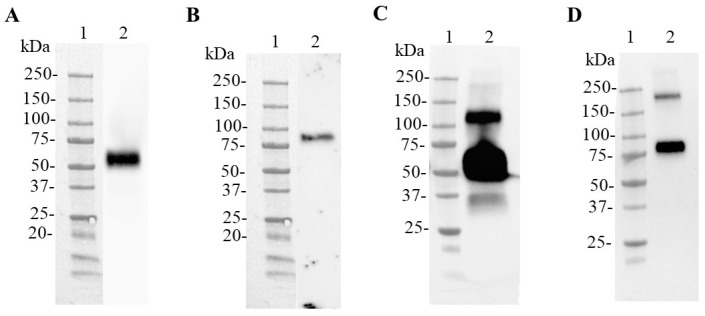
Analysis of the SARS-CoV-2 N and N-CD proteins produced in HEK-293SF-3F6 cells using shake flasks. Western blot of culture supernatant of cells transfected to express the N protein (**A**,**C**) or the N-CD protein (**B**,**D**). HEK-293SF cells were transfected in 1 L shake flasks using HyCell TransFx-H medium. Seventy-two hours post transfection, the cell culture supernatant was collected. Equal volumes of 5-fold (**A**,**C**,**D**) or 11-fold (**B**) concentrated cell culture supernatant were loaded onto the SDS-PAGE gel under reducing conditions. Amicon^®^ Ultra-2 mL centrifugal filters were used for concentration. For (**A**,**B**), a 6x-His Tag monoclonal antibody was used for detection. For (**C**,**D**), a SARS-CoV-2 N protein mouse monoclonal antibody was used as primary antibody. (**A**,**C**), Lane 1: Precision Plus ProteinTM All Blue Prestained Standard, lane 2: cell culture supernatant of cells transfected to express the N protein. (**B**,**D**) Lane 1: Precision Plus ProteinTM All Blue Prestained Standard, lane 2: cell culture supernatant of cells transfected to express the N-CD protein. Protein visualization was carried out with ECL detection system.

In order to obtain N and N-CD proteins under the same conditions and with more integrity, both proteins were produced in HEK-293SF-3F6 cells through transient expression in suspension conditions and serum-free medium (HyCell TransFx-H medium). As a first approach, PEIPro^®^ transfection reagent was used to transfect cells. The presence of the proteins of interest in the cell culture supernatant was confirmed with Western blot analysis using samples collected at 72 h post transfection (Figure 2). In this experiment, the 6x-His Tag monoclonal antibody detected one band with an electrophoretic migration between 50 and 75 kDa according to the molecular weight marker used in the sample from cells transfected to produce the N protein (Figure 2A). This antibody also recognized one band between 75 and 100 kDa in the cell culture supernatant related to N-CD protein production (Figure 2B). The blot showed differences in expression levels, because the N protein was clearly detected when the supernatant was 5-fold concentrated, while the N-CD protein was only observed when it was 11-fold concentrated. No lower molecular weight bands were observed. These results were confirmed using an anti-SARS-CoV-2 N-protein monoclonal antibody (Figure 2C,D). In the case of the N protein, a wide, intense protein band between 50 and 75 kDa and another band between 100 and 150 kDa were observed. The analysis of the N-CD samples showed the expected band between 75 and 100 kDa and, additionally, the antibody recognized a band between 150 and 250 kDa, suggesting the formation of higher molecular weight structures.

### 3.3. Production of the SARS-CoV-2 N and N-CD Proteins in 1 L and 3 L Stirred-Tank Bioreactors

**Figure 3:**
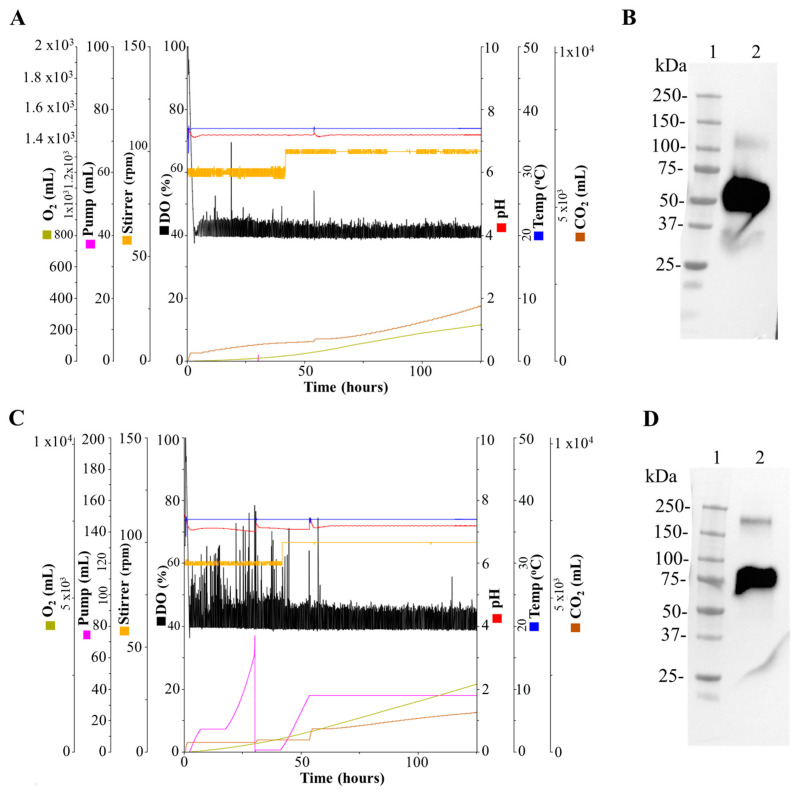
Production of the SARS-CoV-2 N and N-CD proteins in 1 L bioreactors. HEK-293SF-3F6 cells were transfected with linear PEI (MW 25,000) in HyCell TransFx-H medium with the plasmid pl6twblast-N-His or pl6twblast-N-CD. Injected O_2_ and CO_2_, base pumping (pump), agitation rate (stirrer), dissolved oxygen (DO), pH and temperature were monitored during the run before and after the transfection to produce the N protein (**A**) or the N-CD protein (**C**). Western blot of culture supernatant of cells transfected to express the N protein (**B**) or the N-CD protein (**D**). Seventy-two hours post transfection, the cell culture supernatant was collected and concentrated with Amicon^®^ Ultra-2 mL centrifugal filters. Equal volumes of 5-fold concentrated cell culture supernatant were loaded onto the SDS-PAGE gel under reducing conditions. SARS-CoV-2 N protein monoclonal antibody was used as primary antibody. (**B**) Lane 1: Precision Plus ProteinTM All Blue Prestained Standard, lane 2: cell culture supernatant of cells transfected to express the N protein. (**D**) Lane 1: Precision Plus ProteinTM All Blue Prestained Standard, lane 2: cell culture supernatant of cells transfected to express the N-CD protein. Protein visualization was carried out with an ECL detection system.

Several parameters were monitored during the run for both 1 L bioreactors before and after transfection of HEK-293SF-3F6 cells with linear PEI (MW 25 kDa) in HyCell TransFx-H medium. The data showed a good performance during the runs to produce the N and N-CD proteins (Figure 3A,C). Transfection was carried out at 48 h post seeding (cell density at seeding of 0.35 × 10^6^ cells/mL). At that time, Bioreactor 1 (N protein) resulted in values of 1.15 × 10^6^ cells/mL (96.5% of cell viability) and Bioreactor 2 (N-CD protein) had a cellular concentration of 1.37 × 10^6^ cells/mL (97.2% of cell viability). Seventy-two hours post transfection, the cell concentration values were slightly higher with a decrease in cell viability: 73.9% and 82.2% for Bioreactor 1 (N protein) and Bioreactor 2 (N-CD protein), respectively. Analysis with Western blot of the cell culture supernatant 72 h post transfection using the anti-SARS-CoV-2 N-protein monoclonal antibody showed no evident protein degradation and a higher molecular weight band in both cases (Figure 3B,D).

**Figure 4:**
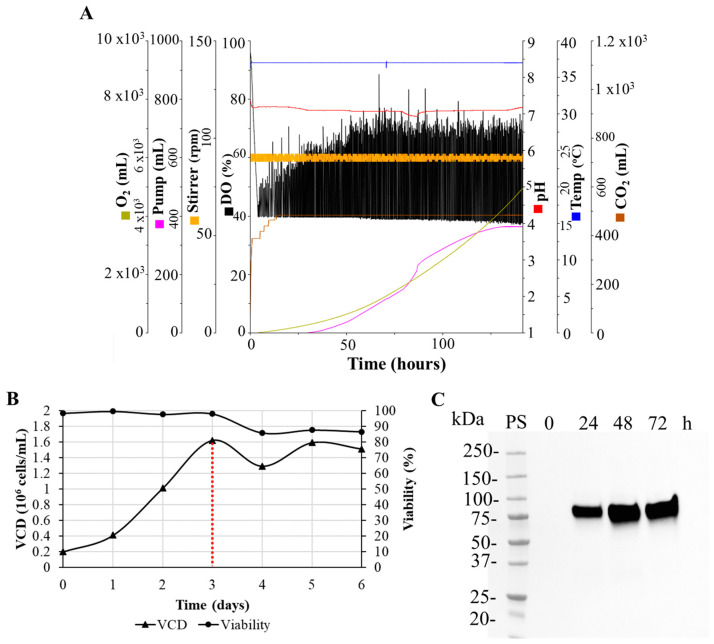
Analysis of growth and expression kinetics of the N-CD protein produced in the 3 L bioreactor. (**A**) Graphic representation of injected O_2_ and CO_2_, base pumping (pump), agitation rate (stirrer), dissolved oxygen (DO), pH and temperature monitored during the run. (**B**) Growth curve of HEK-293SF-3F6 cells cultured in HyCell TransFx-H medium in batch mode. Every 24 h, samples of cell culture were collected and viable cell density (VCD) and cell viability were measured. Cells were transfected with the plasmid pl6twblast-N-CD using linear PEI (MW 25,000). The transfection day is indicated with a red dotted line. (**C**) Samples of cell culture supernatant were collected before transfection (time 0 h) and every 24 h after transfection. Sample concentration was carried out in Amicon^®^ Ultra-2 mL centrifugal filters. Equal volumes of 5-fold concentrated cell culture supernatant were loaded onto the SDS-PAGE gel under reducing conditions. A SARS-CoV-2 N protein mouse monoclonal antibody was used as primary antibody. Precision Plus ProteinTM All Blue Prestained Standard was used as protein standard (PS). Protein visualization was carried out with an ECL detection system.

Due to the lower expression levels of the N-CD protein, another run in a 3 L bioreactor was performed to obtain higher amounts of protein. The in-line monitoring of process parameters was performed (Figure 4A). As we observed in 1 L bioreactors, after transfection the cell viability decreased, with values between 85 and 90% in the first 24 h post transfection. Then, they reached similar values to the transfection day, remaining relatively constant during the last 48 h (Figure 4B). The expression kinetics were analyzed with Western blot. The anti-SARS-CoV-2 N-protein monoclonal antibody detected a band between 75 and 100 kDa corresponding to the N-CD protein, the same as at 24 h post transfection. The peak of expression was reached 48 h post transfection and no protein degradation was detected (Figure 4C).

### 3.4. Purification of the SARS-CoV-2 N and N-CD Proteins Produced in Suspension Culture and Serum-Free Medium

**Figure 5:**
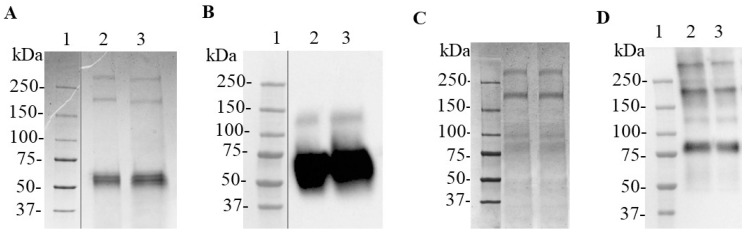
Analysis of the downstream processing of the SARS-CoV-2 N and N-CD proteins produced in suspension culture and protein-free medium. Equal volumes of samples were analyzed under reducing conditions. For protein detection in Western Blot, an anti- SARS-CoV-2 N monoclonal antibody was used as primary antibody. A and B Analysis with Coomassie-stained SDS-PAGE and Western blot of the N protein in formulation buffer, respectively. Lane 1: Precision Plus ProteinTM Unstained Standard (**A**) or Precision Plus ProteinTM All Blue Prestained Standard (**B**), lane 2: N protein after buffer exchange to formulation buffer, lane 3: Main peak of elution fraction at 250 mM imidazole. (**C**,**D**) Analysis with Coomassie-stained SDS-PAGE and Western blot of the N-CD protein in formulation buffer, respectively. Lane 1: Precision Plus ProteinTM Unstained Standard (**C**) or Precision Plus ProteinTM All Blue Prestained Standard (**D**), lane 2: Main peak of elution fraction at 250 mM imidazole, lane 3: N-CD protein after buffer exchange to formulation buffer. For Western blot, protein visualization was carried out with an ECL detection system.

Purification of N and N-CD from the cell culture supernatant was carried out with IMAC after concentration and buffer exchange to reduce the column loading time and the Ni stripping from the resin. The downstream processing was performed in different batches until all the sample was purified. Both proteins eluted at 250 mM imidazole. N protein was desalted to the formulation buffer using Amicon centrifugal filters. N-CD was desalted to the formulation buffer using a PD-10 column, since the buffer exchange and concentration steps in Amicon centrifugal filters resulted in complete loss of the antigen, possibly due to aggregation. Purified proteins were analyzed with SDS-PAGE and Western blot (Figure 5). Protein purity after IMAC purification and buffer exchange was approximately between 50 and 70%.

### 3.5. Obtaining N-CD-Expressing HEK-293 Cell Clones and Adaptation to Serum-Free Medium and Suspension Culture Conditions

**Figure 6:**
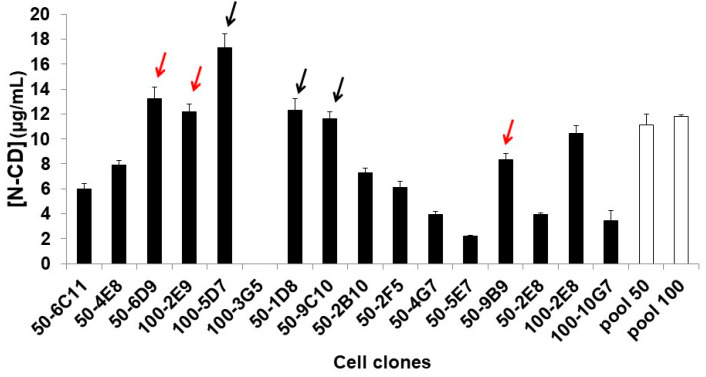
Evaluation of protein expression in N-CD-expressing cell clones generated via limiting dilution cloning from N-CD-expressing HEK-293 cell pools (MOI 50 and 100). The cells were seeded in 24-well plates in DMEM/F12-FBS at 0.3 × 10^6^ cells/well in triplicate. After 7 days, cell culture was harvested and N protein was measured with ELISA. The data correspond to mean ± standard deviation. The white bars correspond to the two different N-CD-expressing HEK-293 cell pools and the black bars to the cell clones derived from these cell pools. The six arrows point out the cell clones selected for adaptation to protein-free and suspension culture conditions in CDM4HEK293 medium. The red arrows indicate the cell clones that survived to the adaptation process.

Sixteen N-CD-expressing HEK-293 stable cell clones were obtained by limiting dilution cloning in adherent conditions and serum-supplemented medium. N-CD expression levels were between 2 and 18 µg/mL with ELISA (Figure 6). The cell clones with higher or similar expression levels to the parental N-CD-expressing HEK-293 cell pools (MOI 50 and 100) were selected for adaptation to serum-free and suspension culture conditions in CDM4HEK293 medium. The selected cell clones were 50-1D8, 50-6D9, 50-9B9, 50-9C10, 100-2E8 and 100-2E9. However, only three cell clones (50-6D9, 50-9B9 and 100-2E9) survived to the adaptation process. 

**Figure 7:**
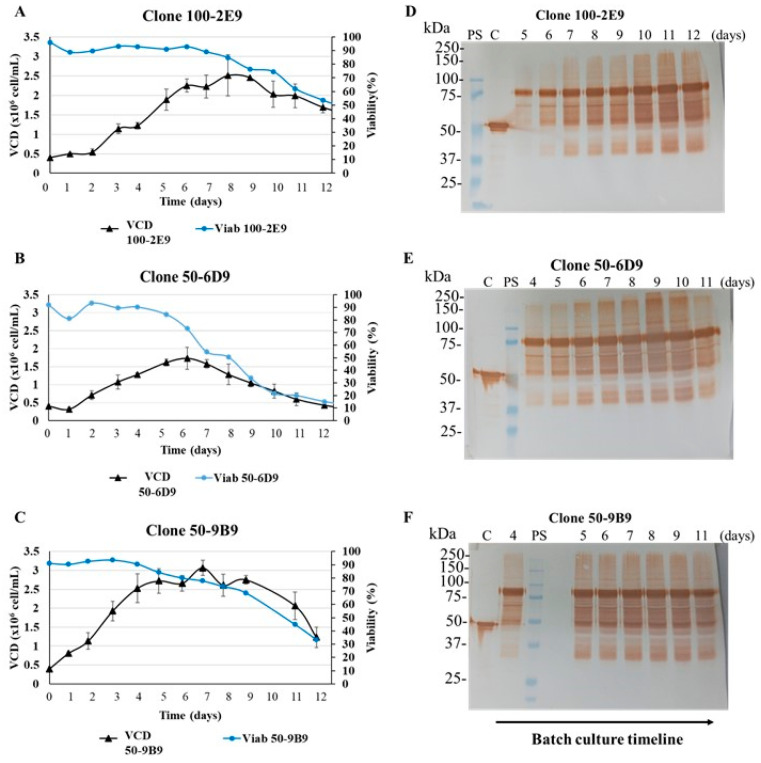
Batch culture of N-CD-expressing cell clones adapted to protein-free medium and suspension culture conditions. (**A**–**C**) Growth profile of cell clones 100-2E9, 50-6D9 and 50-9B9, respectively. The cells were seeded in 250 mL shake flasks at 0.4 × 10^6^ cells/mL in 90 mL of CDM4HEK293 medium. The shake flasks were set in a shaker (130 rpm) and incubated in a warm chamber (37 °C) without CO_2_. The experiment was performed in duplicate. Every 24 h, samples of cell culture were collected and viable cell density (VCD) and cell viability were measured. After sampling, the flasks were incubated at 37 °C with 5% CO_2_ in static conditions for 2 or 3 h, and, later, they were incubated again in the conditions described above. (**D**–**F**) Analysis with Western blot of cell culture supernatant of cell clones 100-2E9, 50-6D9 and 50-9B9, respectively. Total proteins from equal volumes of cell culture supernatant, precipitated by the methods of TCA and sodium deoxycholate, were loaded onto the SDS-PAGE gel under reducing conditions. The supernatant samples analyzed corresponded to the exponential and stationary growth phases. The specific days analyzed are pointed out in the figure. For protein detection, the membrane was exposed to an HRP-conjugated anti-SARS-CoV-2 N-protein monoclonal antibody. PS: Precision Plus ProteinTM All Blue Prestained Standard was used as protein standard. C: SARS-CoV-2 N protein expressed in *E. coli* was used as positive control. Protein visualization was carried out with DAB.

The three surviving clones showed different behaviors in batch culture using shaking flasks in CDM4HEK293 medium and agitated conditions (Figure 7A–C). Table 1 summarizes the growth characteristics of these three clones in batch culture. 

The cell clone 50-9B9 showed higher maximum values of VCD, IVCC and μmax than the other cell clones. The cell clone 50-6D9 showed the poorest growth profile, with the lowest maximum values of VCD and IVCC. The cell clone 100-2E9 presented similar values of VCD and IVCC than this last clone and a cell viability above 90% for 6 days, as a main advantage. The other two cell clones only showed a cell viability higher than 90% for 4 days. The N-CD expression levels were not measured with ELISA, due to the loss of sensitivity of this technique when samples from cells cultured in CDM4HEK293 medium were processed. The presence of the N-CD protein in the cell culture supernatant from batch cultures was confirmed with Western blot under reducing conditions (Figure 7D–F). The anti-SARS-CoV-2 N-protein monoclonal antibody detected one band between 75 and 100 kDa with high intensity, which corresponds to the N-CD protein. Other bands (approximately 50 kDa and 37–25 kDa) were also recognized, with a lower intensity. 

### 3.6. Batch Culture of the N-CD-Expressing HEK-293 Cell Clone 100-2E9 and Simulation of Perfusion Conditions

**Figure 8:**
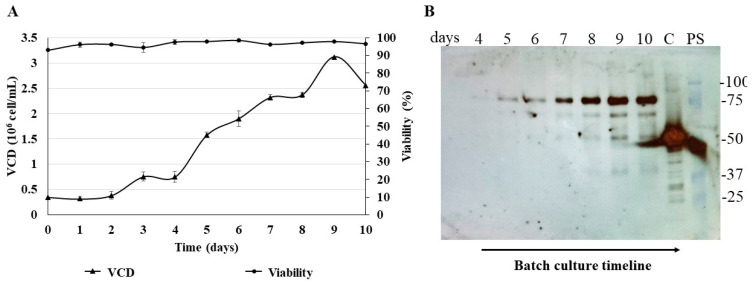
Batch culture of the N-CD-expressing cell clone 100-2E9 in 2 L roller bottles. (**A**) Growth profile of cell clone 100-2E9 in protein-free medium and suspension culture conditions. The 2 L roller bottles were seeded at 0.35 × 10^6^ cells/mL in 500 mL of CDM4HEK293 medium and placed vertically in an incubator (Infors HT, Switzerland) at 37 °C with 5% CO_2_ in shaking conditions (120 rpm). The experiment was performed in duplicate. Every 24 h, samples of cell culture were collected and viable cell density (VCD) and cell viability were measured. (**B**) Analysis with Western blot of cell culture supernatant. Total proteins from equal volumes of cell culture supernatant, precipitated by the methods of TCA and sodium deoxycholate, were loaded onto the SDS-PAGE gel under reducing conditions. The analyzed supernatant samples correspond to the exponential growth phase. For protein detection, the membrane was exposed to an HRP-conjugated anti-SARS-CoV-2 N-protein monoclonal antibody. C: SARS-CoV-2 N protein expressed in *E. coli* (45 kDa) was used as positive control. PS: Precision Plus ProteinTM All Blue Prestained Standard was used as protein standard. Protein visualization was carried out with an ECL detection system.

Due to the better cell growth performance of the N-CD-expressing HEK-293 cell clone 100-2E9, this clone was selected for further experiments. A batch culture study was repeated using 2 L roller bottles (Figure 8A). A similar μ_max_ was obtained, but the maximum viable cell density showed a slightly higher value (3.1 × 10^6^ cell/mL) compared with the previous batch cultured in 250 mL shake flasks (Table 1). Cell viability was above 90% during the 10 days of this experiment. The IVCC_max_ showed a lower value because it was calculated taking into account the data obtained at the end of the experiment. The batch culture in the 2 L roller bottles lasted only 10 days in comparison with the batch culture in the 250 mL shake flasks (16 days). On day 10, the IVCC in the 250 mL shake flasks was 376.5 × 10^8^ cells × h/mL. A similar value was obtained in the 2 L roller bottle experiment. The Western blot analysis under reducing conditions showed, again, one main band between 75 and 100 kDa in the cell culture supernatant (Figure 8B). 

**Figure 9:**
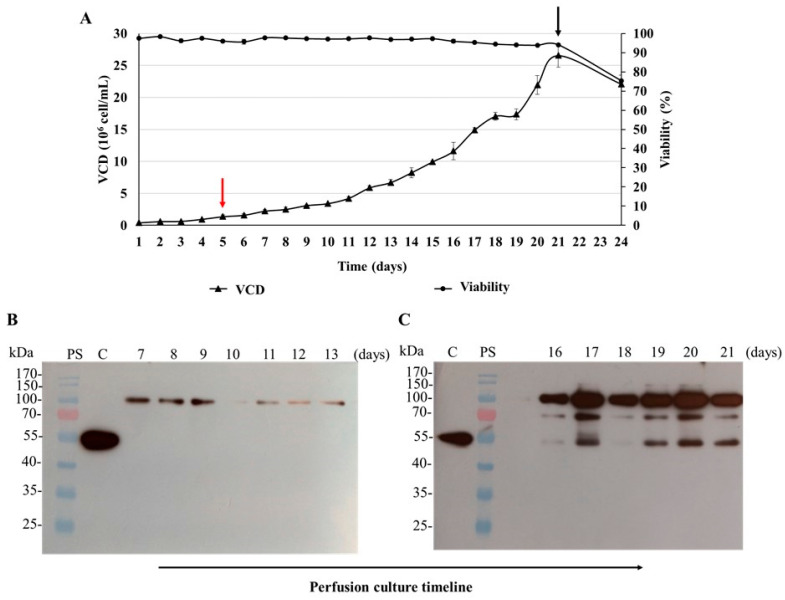
Simulation of perfusion culture of the N-CD-expressing cell clone 100-2E9 in shake flasks. (**A**) Growth profile of cell clone 100-2E9 in protein-free medium and suspension culture conditions. The 250 mL shake flasks were seeded at 0.4 × 10^6^ cells/mL in 90 mL of CDM4HEK293 medium and incubated at 37 °C with 5% CO_2_ in shaking conditions (120 rpm). The experiment was performed in duplicate. The cells were maintained in batch culture conditions until the exponential phase and maximum growth rate were reached. The CSPR was fixed to 0.15 nL/cell/day. Every 24 h, samples of cell culture were collected. Viable cell density (VCD) and cell viability were measured. At the end of perfusion culture, the cells were grown in batch culture for 3 days. The beginning and the end of the perfusion culture are indicated with red and black arrows, respectively. (**B**) Analysis with Western blot of cell culture supernatant. Total proteins from equal volumes of cell culture supernatant, precipitated by the methods of TCA and sodium deoxycholate, were loaded onto the SDS-PAGE gel under reducing conditions. The analyzed supernatant samples correspond to the exponential growth phase; the specific analyzed days are pointed out in the figure. For protein detection, the membrane was exposed to an HRP-conjugated anti-SARS-CoV-2 N-protein monoclonal antibody. PS: Page RulerTM Prestained Protein Ladder was used as protein standard. (**C**) SARS-CoV-2 N protein expressed in *E. coli* (45 kDa) was used as positive control. Protein visualization was carried out with an ECL detection system.

The next step was to assess the performance of the cell clone 100-2E9 at high density and perfusion-like culture (Figure 9A). Cells were cultured in CDM4HEK293 medium using shaking flasks and agitated conditions. The shaking flasks were inoculated and 5 days later the simulation of perfusion started with an average dilution rate of 0.23 vvd until reaching 1 vvd. The exponential phase of cell growth continued for 17 days, and 27 × 10^6^ cells/mL was the maximum viable cell density reached in this experiment. The cell viability was always above 90%. During the run, µ was around 0.019 h^−1^. The cell culture supernatant was analyzed with Western blot under reducing conditions and a major band between 75 and 100 kDa was observed. Other bands (approximately 70 and 55 kDa) were also detected by the anti-SARS-CoV-2 N-protein monoclonal antibody in samples from days 16 to 21, with lower intensity (Figure 9B,C).

## 4. Discussion

Previous work demonstrated that the N-CD protein induced a high and long-lasting N-specific IgG response with only two doses in pre-clinical trials, which made this protein a promising vaccine antigen against SARS-CoV-2 [11]. However, the protein degradation observed in adherent HEK-293 cell cultures in that study threatened its conception as a final product. The N-CD protein has an expected molecular weight of 73.1 kDa; however, the anti-SARS-CoV-2 N-protein monoclonal antibody recognized one band between 75 and 100 kDa and three groups of bands with a lower molecular weight (approximately 75–50, 50 and 37–25 kDa) under reducing conditions. At that moment, we hypothesized that proteases present in the serum used to supplement the culture medium could degrade the N-CD protein [14,15]. Although the cell monolayer was rinsed several times with PBS before adding the serum-free medium for harvesting, some proteases can remain and cut the N-CD protein. Therefore, culturing the cells in suspension culture and serum-free media could be one approach to overcome or improve protein degradation. 

In this work, we found that the N protein produced in adherent conditions and serum-supplemented medium showed a consistent pattern of degradation similar to that observed in the N-CD protein. This result confirmed that the SARS-CoV-2 N protein was susceptible to being degraded in those culture conditions and the degradation of the N-CD protein was related to the N portion of this chimeric protein. 

The SARS-CoV-2 N protein has mainly been produced in *E. coli*. There are at least 42 scientific publications related to this topic [31,32,33,34,35,36,37,38,39,40,41,42,43,44,45,46,47,48,49]. The other expression system most used for this purpose is mammalian cells, specifically HEK-293 [19,20,50,51,52,53,54,55,56,57] and CHO cells [58,59], with intracellular and/or extracellular protein expression. Few examples were found related to N-protein production in plant (*Nicotiana benthamiana*) [39,60,61] and insect cells [62,63]. An analysis of the results published demonstrated that in several cases the N protein showed lower molecular bands probably caused by degradation, similar to those observed in our work. This phenomenon was seen in the four expression systems: *E. coli* [34,38,39,42,43,44,45,46,47,48,49], mammalian cells [19,20,51,58,59], plants [39,60,61] and insects [63]; it was more obvious in eukaryotics. However, most of them did not mention and none of them explained the degradation pattern of the expressed SARS-CoV-2 N protein. 

A comprehensive analysis of the degradation of the SARS-CoV-2 N protein expressed in *E. coli* was recently studied by Lutomski et al. [64]. The authors found that the N protein undergoes autoproteolysis at highly conserved residues in the vicinity of the linker region to generate at least five unique proteoforms (22.6 kDa, 23.5 kDa, 28.7 kDa, 29.4 kDa and 42.9 kDa). They also identified various stoichiometries of N proteoforms that were influenced by pH. The molecular weight of these five proteoforms is in agreement with the mass of the lower molecular weight bands observed in HEK-293 cells cultured in adherent conditions, taking into account a mass shift due to the protein glycosylation that takes place in mammalian cells [65,66]. Indeed, the glycosylation of the SARS-CoV-2 N protein has been reported in other studies [51,52]. These autoproteolytic properties have also been identified for the SARS-CoV N protein [67]. In a mass spectroscopy study of the SARS-CoV N protein produced in *E. coli*, the authors found that the extra bands observed (29 and 25 kDa) were proteolytic products of the N protein, and the cleavage appeared to be due to inherent instability and/or autolysis [67]. Other authors have proposed additional mechanisms to explain the degradation of the N protein, such as cleavage by caspases [68,69]. 

Despite the cell monolayer being rinsed with PBS for harvesting in transient experiments in adherent conditions, the long-time culture in starving conditions for cells non-adapted to serum-free medium could probably be another factor that stressed the cells and contributed to cell death and the release of proteases (i.e., caspases) into the cell culture supernatant. Moreover, the accumulation of toxic waste products over time can favor an environment that promotes the N protein’s autocatalytic activity [70,71]. The growth kinetic experiment with both N-protein-expressing pools simulating the harvesting procedure in 24-well plates showed that protein degradation was present even after 24 h of being added to the medium without FBS. In addition, at least 5 to 6 days were needed to allow for the accumulation of the corrected size protein in the cell culture supernatant. Producing the N and N-CD proteins in suspension cultures and serum-free media constitute an alternative to obtain high-quality proteins, taking into account that the adherent culture and harvesting conditions were not ideal to avoid N protein degradation. The adaptation of stable recombinant HEK-293 cells to those conditions is a complex process that needs several months to be completed; thus, the transient expression in HEK-293 cells in suspension with serum-free media constitutes a suitable alternative to assess how these culture conditions can improve the quality of both N and N-CD proteins. Indeed, the results showed that both proteins produced in these conditions were not degraded. This observation was confirmed in experiments with cells cultured in shaken flasks to 1 L and 3 L bioreactors. In addition, the transient expression procedures performed in this work allowed us to produce, in a shorter time (1–2 weeks), enough protein for pre-clinical studies in a laboratory set-up closer to a final industrial production setting. Working in suspension conditions enabled an easier assessment of the manufacturing process in 1 L and 3 L bioreactors, which is a guarantee of potential scalability to larger volumes, unlike in the adherent conditions. The cell culture scale-up of adherent cells is to a certain extent limited by culture surface availability and brings its own set of challenges, such as costly culture flasks and extensive manipulations [72,73,74]. On the other hand, in terms of downstream processing, the use of serum-free medium increases purification efficiency, decreases the overall cost of operations and provides advantages from a regulatory point of view, thus enhancing the production throughput [75] and acceptability of the final product.

For the optimal transient expression at a larger scale of the N and N-CD proteins, several aspects were taken into account, such as the cell line, the transfection reagent and the culture medium [76]. First, we used an HEK-293 suspension cell line, which is widely used for recombinant protein and viral vector production as it offers many advantages related to high transfection yields, is easily grown in suspension culture and adapts to several commercially available serum-free media [72,73,76,77,78]. Specifically, our experiments were performed in an HEK293 serum-free cell line derived from clone HEK-293SF-3F6. This clone was isolated and selected for its ability to grow at the highest specific cell growth rate and cell density in suspension culture [18,26,75]. Second, the transfection reagent should be cost-effective, simple to use, effective in cells growing in suspension culture and have minimal cytotoxic effects [73,75,76]. We used 25 kDa linear PEI as the transfection reagent, which satisfies most of these criteria. Furthermore, it has been successfully implemented in many procedures to transfect suspension-adapted HEK-293 cells from the small- to large-scale [18,22,23,72,75,76,79,80,81]. Interestingly, we observed that the growth was almost arrested and cells entered lag phase after the addition of the PEI–plasmid complex to the cells cultured in the 3 L bioreactor to produce the N-CD protein. This lag phase was probably caused by the stress induced by the transfection [75]. Although the cell viability decreased after this step, the values were between 85 and 90%, showing that 25 kDa linear PEI only induced slight cytotoxic effects. This growth arrest, after the transfection with 25 kDa linear PEI of HEK-293 cells cultured in suspension conditions in bioreactors, has also been reported by other authors [18,75]. Third, we carried out transfection, growth and expression in Hyclone HyCell TransFx-H [18,81], a versatile culture medium that eliminates the need for medium exchange prior to and/or after transfection, which is a bottleneck to overcome in large-scale transient expression [80]. Therefore, the same culture medium was used during the complete process, resulting in a simpler procedure than those requiring a switch in media for transfection [25]. Another key point for the optimal large-scale transient transfection is the expression vector [21,76]. We did not optimize expression vectors in this work; however, this can be performed in future experiments to optimize protein expression. 

Other recombinant proteins have been produced in similar conditions as those described in this work, using HEK-293 cells cultured in suspension, serum-free media and 25 kDa linear PEI as the transfection agent. The expression levels in the cell culture supernatant ranged from 1 to 1000 mg/L for the production of SARS-CoV-2 RBD [18], IgG [72,79,80,81], Fc-fusion proteins to the soluble receptors [79], secretory glycoproteins [23] and other complex proteins [22,77]. 

Another important consideration was the presence of high molecular aggregates in the samples analyzed with Western blot even in reducing conditions. The structure of coronavirus N proteins presents two conserved and independently folded structural domains, called the N-terminal domain (NTD) and C-terminal domain (CTD) [82,83]. Previous studies have revealed that the N-NTD is responsible for RNA binding and the N-CTD for both RNA binding and dimerization [82,83]. Three different crystallographic studies of the SARS-CoV-2 N-CTD structure revealed a compact, tightly intertwined homodimer with an overall rectangular slab shape [83,84,85]. In the samples related to N-protein expression, the anti-SARS-CoV-2 N monoclonal antibody recognized a band between 50 and 100 kDa and another between 100 and 150 kDa, corresponding to the molecular weight for monomer and dimer, respectively. In the case of the N-CD protein samples, the same antibody detected three bands in molecular weight intervals of 75–100 kDa, 150–250 and higher than 250 kDa, probably consistent with the monomer, dimer and trimer of this protein, respectively. The property of the N-CD protein to form trimers is probably related to the CD154 fragment. CD154 is a type II membrane glycoprotein with a cytoplasmic tail, a transmembrane domain and an extracellular domain in humans [86,87]. Similar to other members of the tumor necrosis factor (TNF) superfamily, CD154 contains a conserved TNF-homology domain in the extracellular region that enables trimerization and receptor binding. CD154 assembles as noncovalent homotrimers or heterotrimers consisting of full-length and truncated forms of CD40L [86,87]. A soluble form of CD154 (sCD154) co-exists with its membrane (mCD154). sCD154 can be generated with alternative splicing in the cytoplasm or from a proteolytic process [87]. These shorter soluble forms of CD154 retain their ability to form trimers, to bind CD40, and to deliver biological signals, thus indicating that CD154 might also act as a bona fide cytokine [86,88]. Moreover, CD40/CD154 interaction has been analyzed through molecular modeling, biochemical and biophysical analyses, mutagenesis and X-ray crystallography. The binding of trimeric mCD154 or sCD40L to the CD40 receptor on the cell surface is necessary for the trimerization of the receptor, which is required for downstream signaling [87,89,90]. This evidence from the literature indicates that the trimer formation by the N-CD protein is a consequence of the correct folding of the CD154 portion and, hence, of its capability to bind and activate the CD40 receptor in the effector cells.

The results obtained with Western blot from N-CD-expressing HEK-293 cell clones adapted to suspension culture and serum-free media confirmed the results from transient expression in these culture conditions. The N-CD protein expressed by the adapted recombinant cell clones showed almost no degradation when they were cultured in batch mode at different scales or in perfusion culture at high cellular densities. The adaptation process of the recombinant cell clones took approximately six months and the overall production of the N and N-CD proteins in shake flasks and bioreactors was carried out between 1 and 2 months. There is a clear advantage in the transient expression related to saving time and resources. 

In conclusion, the results support transient expression in suspension culture and serum-free media as a useful tool in the earlier stages of an investigation, when several candidates are tested and the generation of a recombinant and stable cell line is not required. In addition, we demonstrated how the cell culture conditions can dramatically affect the quality of labile proteins such as the SARS-CoV-2 N protein. Protein degradation in a therapeutic product not only impacts the long-term product stability, but it can also potentially compromise patient safety and product efficacy [91,92]. These findings could also be relevant for the N proteins of other coronaviruses. Further experiments will be performed to evaluate and compare how the degradation levels of the N-CD protein, produced in different culture conditions, can affect the immune response against this vaccine candidate.

## Figures and Tables

**Table 1:** Growth characteristics of HEK-293 cell clones in batch culture using CDM4HEK293 medium.

Cell Clones	Growth Phase Duration ^§^ (days)	Experiment Duration (days)	VCD_max_ (10^6^ cell/mL)	IVCC_max_ (10^8^ cells × h/mL)	μ_max_ (h^−1^)
50-6D9 *	4	16	1.7	314.5	0.0116
50-9B9 *	4	13	3.1	632.8	0.0159
100-2E9 *	6	16	2.5	592.7	0.0105
100-2E9 **	10	10	3.1	354.3	0.0101

^§^ Growth phase duration with a cell viability above 90%. VCD_max_: maximum viable cell density. IVCC_max_: time integral of viable cell concentration obtained at the end of the experiment. μ_max_: maximum growth rate. Asterisks indicate that the experiment was performed in 250 mL shake flasks (*) or 2 L roller bottles (**).

## Data Availability

All relevant data are included in the manuscript.

## Supplementary Materials

The following supporting information can be downloaded at: https://www.mdpi.com/article/10.3390/biomedicines11113050/s1, Figure S1: N protein expression in HEK-293 cells in adherent conditions.; Figure S2: Analysis with SDS-PAGE 10% and Western blot of the SARS-CoV-2 N and N-CD proteins produced in HEK-293SF-3F6 cell supernatant using 1 L bioreactors.
