# Supplementary material for: Transient Expression in HEK-293 Cells in Suspension Culture as a Rapid and Powerful Tool: SARS-CoV-2 N and Chimeric SARS-CoV-2N-CD154 Proteins as a Case Study
Source: Biomedicines. 2023 Nov 14;11(11):3050. doi: 10.3390/biomedicines11113050 (PMC10669214; doi:10.3390/biomedicines11113050)

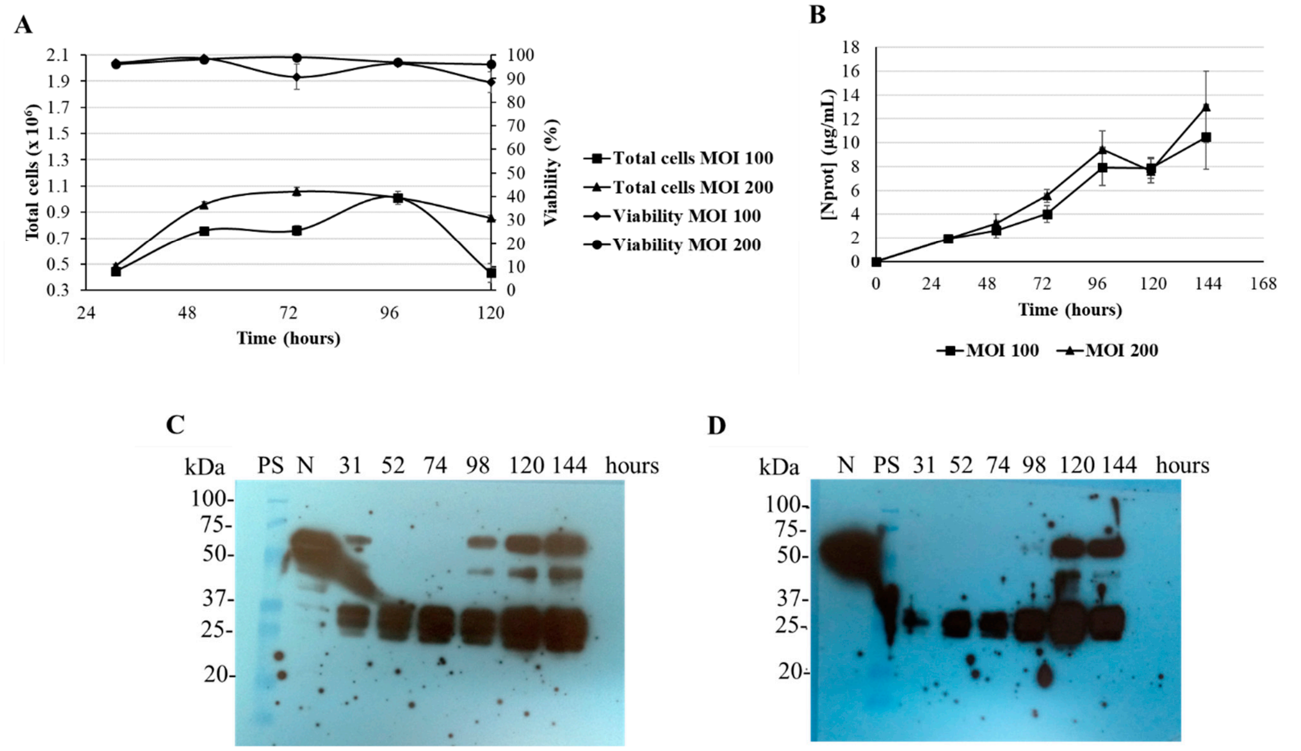

Figure S1 N protein expression in HEK-293 cells in adherent conditions. The recombinant cells were seeded at 0.3 x 10^6 cells per well in 1 mL of DMEM/F12-FBS in triplicate in 24-well plates. Six plates were seeded in the same conditions, one per each day of the kinetic study. Twenty-four hours later, the cell monolayer was carefully washed with PBS and 1 mL of DMEM/F12 medium was added per well. The plates were incubated again at 37 °C in 5% CO_2 and approximately every 24 hours, samples of cell culture were collected and cell density and viability were determined. **A:** Growth profile of HEK-293-N-100 cells and HEK-293-N-200 cells cultured in adherent conditions. **B**: N protein concentration in the cell culture supernatant determined by ELISA. **C** and **D**: SDS-PAGE 10 % and Western blot analysis under reducing conditions of cell culture supernatant collected approximately every 24 hours from HEK-293-N-100 cells and HEK-293-N-200 cells, respectively. SARS-CoV-2 N protein produced in *E. coli* was used as positive control (N). (PS): Precision Plus Protein™ All Blue Prestained Standard. The antibody CBSSNCoV-1-HRP (1:2000) was used for protein detection. Protein visualization was carried out using ECL detection system.

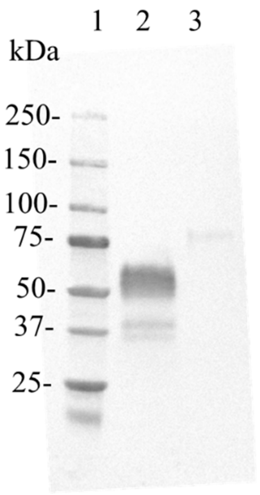

Figure S2 Analysis by SDS-PAGE 10 % and Western blot of the SARS-CoV-2 N and N-CD proteins produced in HEK-293SF cells supernatant using 1 Lbioreactors. In the experiment performed in 1 L-bioreactors, HEK-293SF cells were transfected with linear PEI (MW 25,000) in HyCell TransFx-H medium. Seventy-two h post-transfection the cell culture supernatant was collected and analyzed. Lane 1: Precision Plus Protein™ All Blue Prestained Standard, lane 2: cell culture supernatant of cells transfected to express the N protein, lane 3: cell culture supernatant of cells transfected to express the N-CD protein. Equal volumes of 5-fold concentrated cell culture supernatant were loaded onto the SDS-PAGE gel under reducing conditions. Amicon® Ultra-2 mL centrifugal filters were used for concentration. A 6x-His Tag monoclonal antibody conjugated to horseradish peroxidase (HRP) (His.H8, MA1-21315-HRP, Thermo Fisher Scientific, USA) (1:1000) was used for the detection of the 6x-Histidine tail present in both proteins. Protein visualization was carried out through an ECL detection system.

Original photos for SDS-PAGE and western blots

Figure 1b

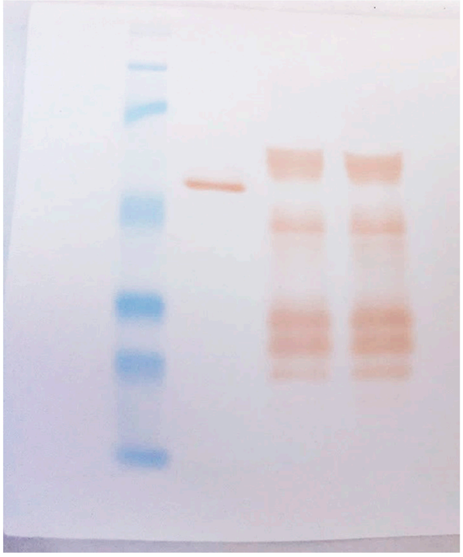

Figure 1c

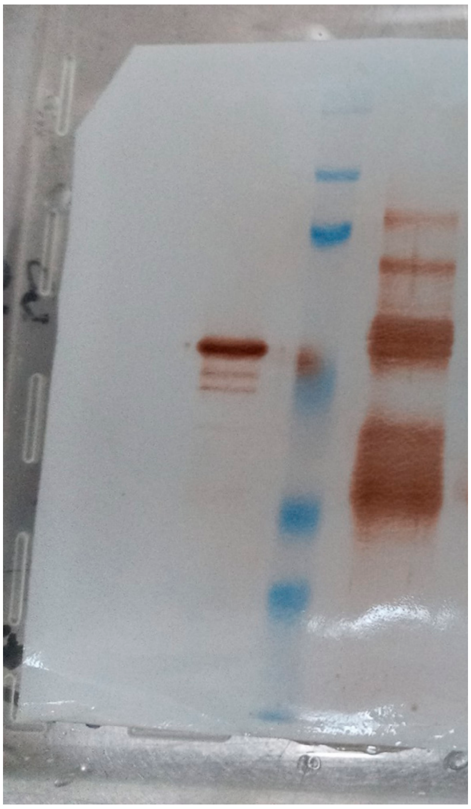

Figure 2a

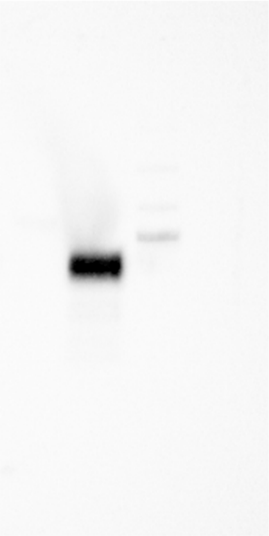

Figure 2b

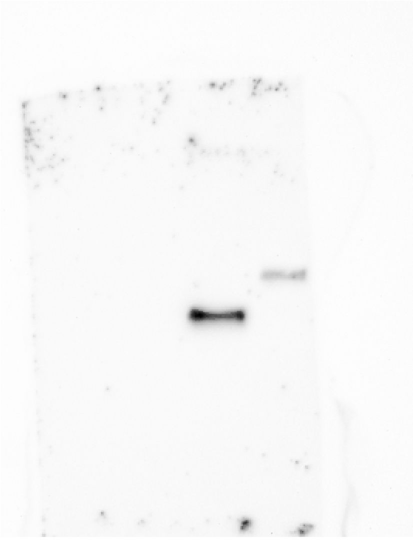

Figure 2c and Figure 3b

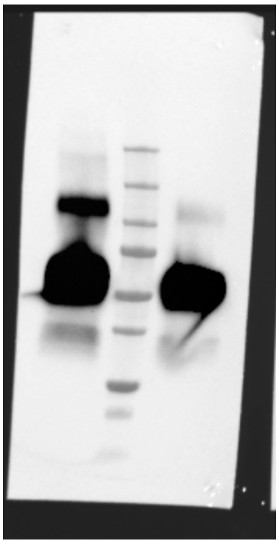

Figure 2d and Figure 3d

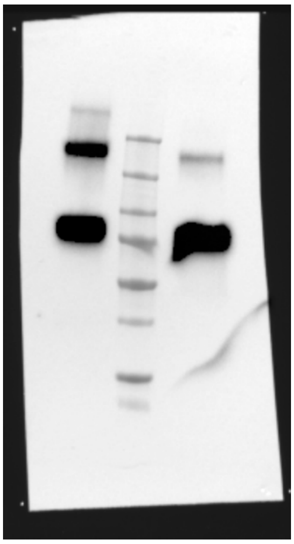

Figure 4c

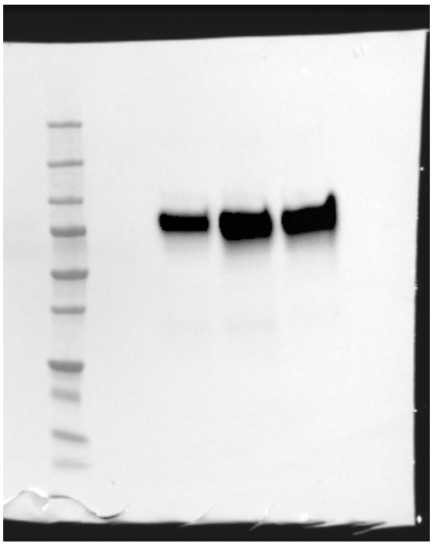

Figure 5a

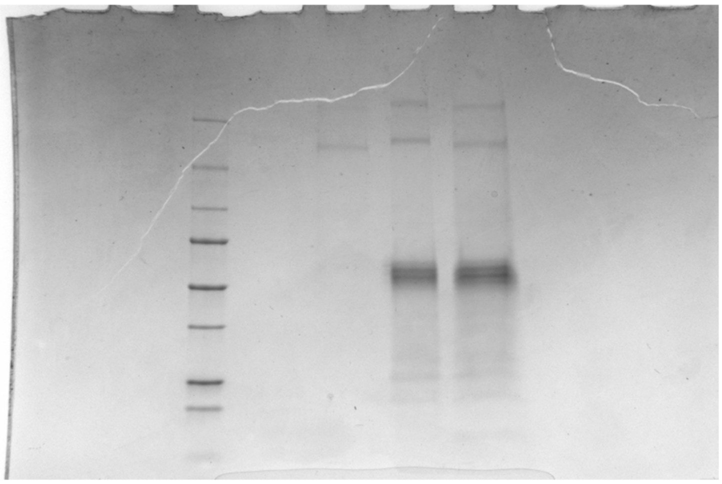

Figure 5b

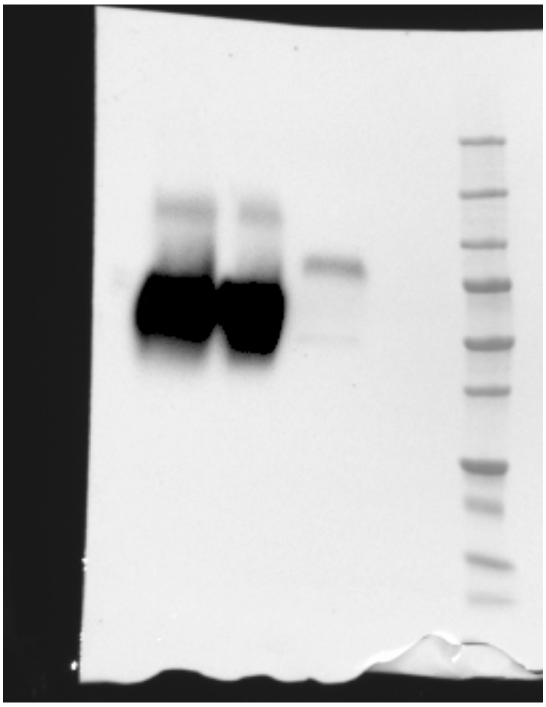

Figure 5c

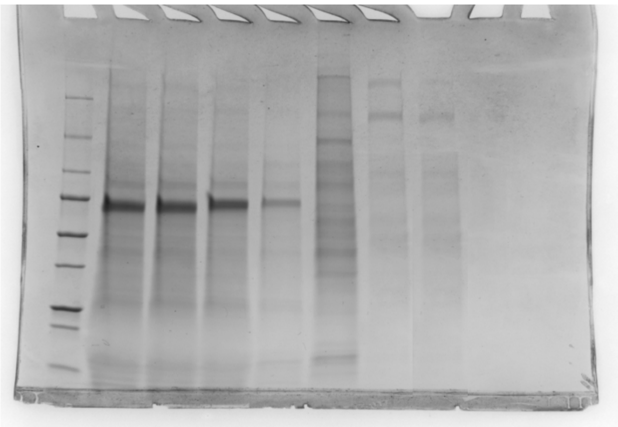

Figure 5d

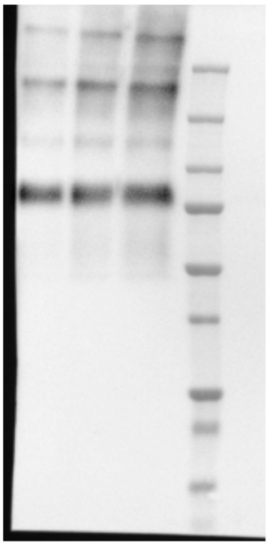

Figure 7d

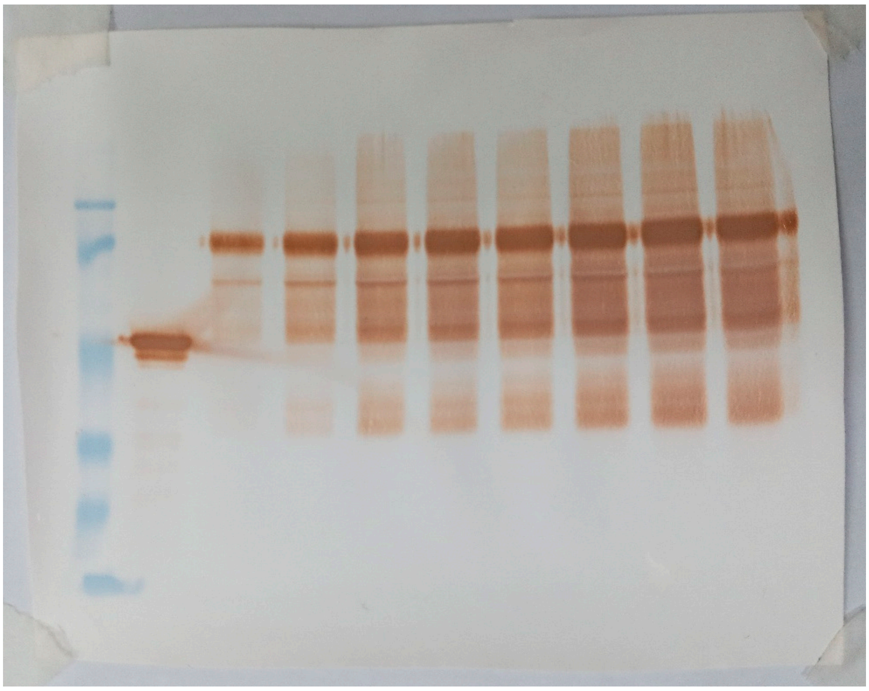

Figure 7e

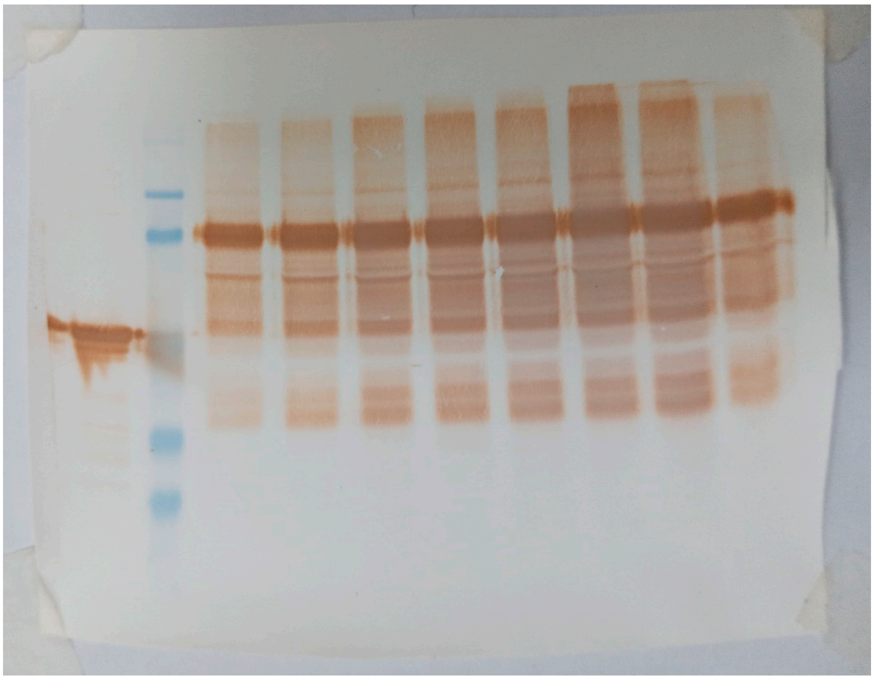

Figure 7f

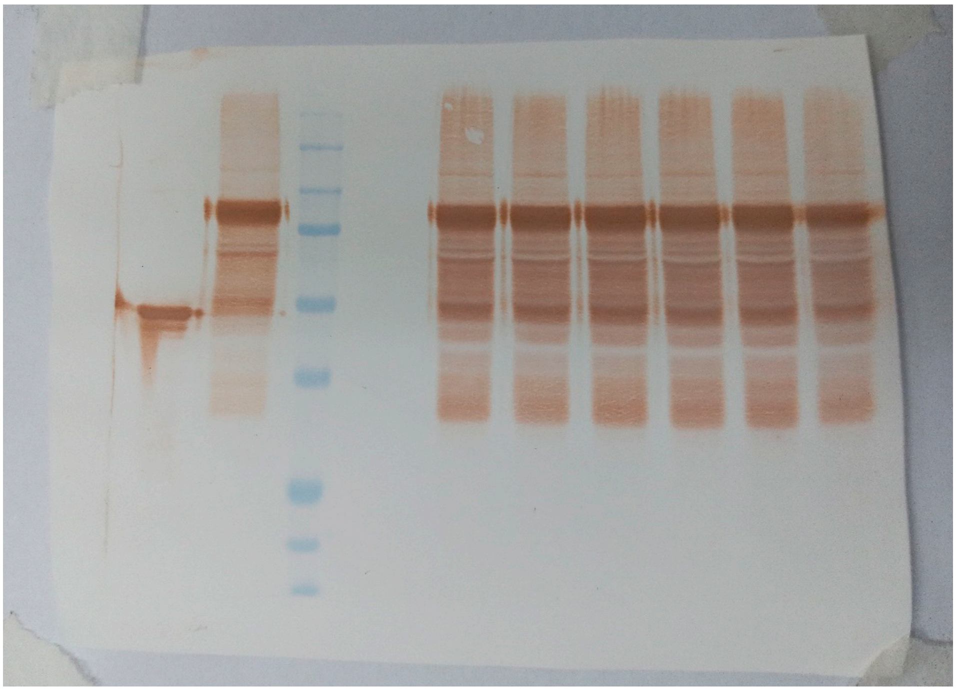

Figure 8b

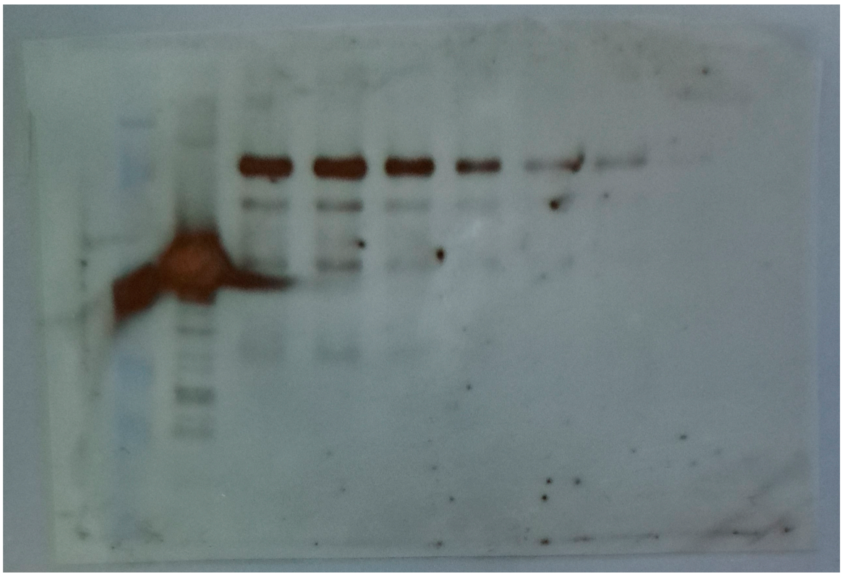

Figure 9b

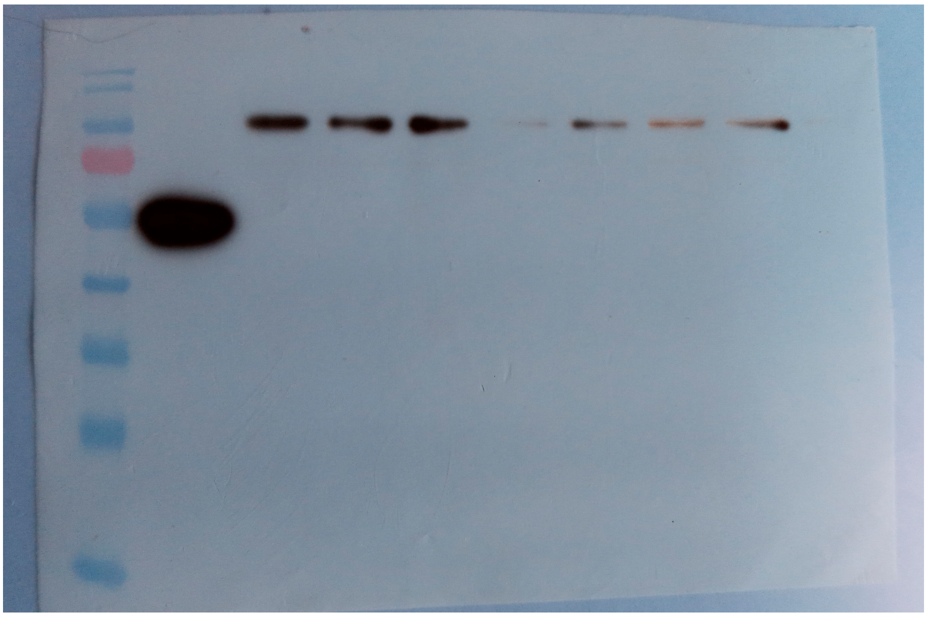

Figure 9c

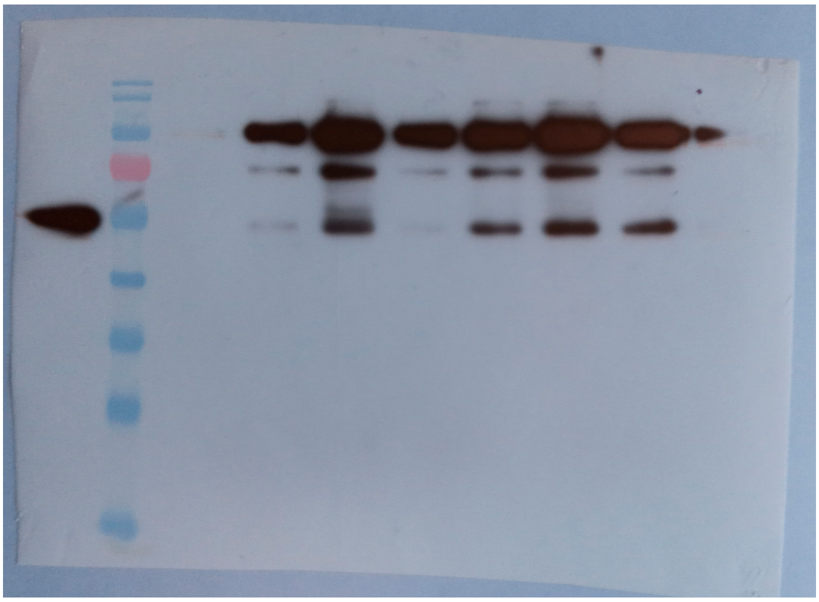

Figure S1c

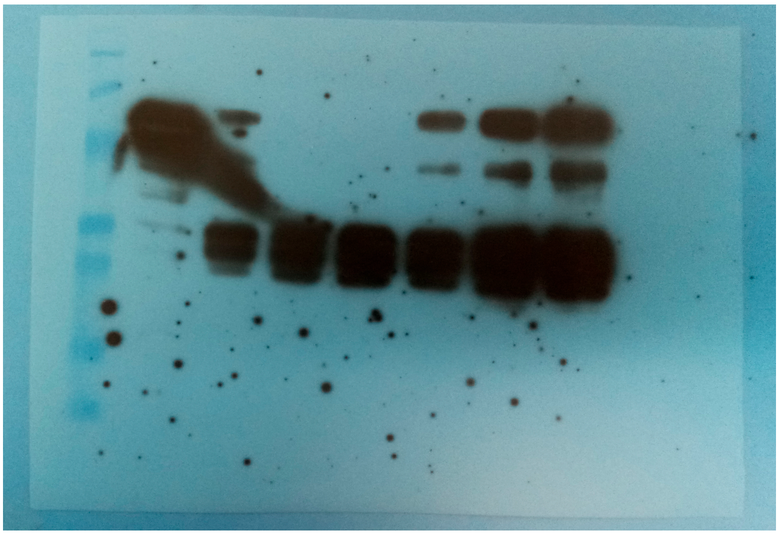

Figure S1d

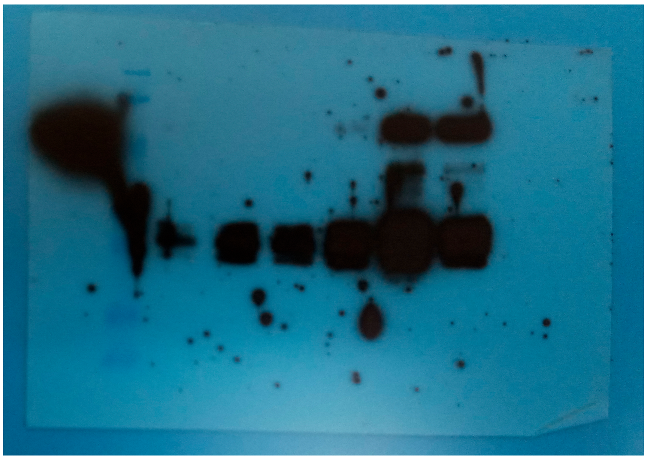

Figure S2

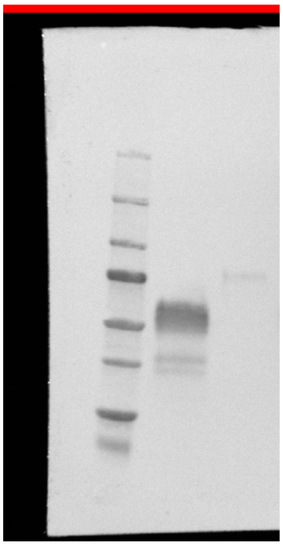

Supplement: Supplementary file 1 [file biomedicines-11-03050-s001.zip › biomedicines-2666160-supplementary.pdf]
